# To Share or Not To Share? Academic Incentives May Hamper Public Good

**DOI:** 10.1021/acs.est.2c05721

**Published:** 2022-10-12

**Authors:** Cesunica E. Ivey, A. Kofi Amegah, Collins Gameli Hodoli, Kerry E. Kelly, Abiola S. Lawal, Pallavi Pant, Saumya Singh, R. Subramanian, Ivette Torres, Daniel M. Westervelt, Haofei Yu

**Affiliations:** † Department of Civil and Environmental Engineering, University of California, Berkeley, Davis Hall, Berkeley, California 94720, United States; ‡ Public Health Research Group, Department of Biomedical Sciences, University of Cape Coast, New Administration Block, Cape Coast, Central RU8217, Ghana; § School of Built Environment, University of Environment and Sustainable Development, Private Mail Bag, Somanya, Eastern Region, Ghana; ∥ Clean Air One Atmosphere, Accra, Ghana; ⊥ Department of Chemical Engineering, University of Utah, 50 South Central Campus Drive, 3290 MEB, Salt Lake City, Utah 84112, United States; # School of Civil and Environmental Engineering, Georgia Institute of Technology, Atlanta, Georgia 30332, United States; @ Health Effects Institute, 75 Federal Street, Suite 1400, Boston, Massachusetts 02110, United States; ∇ Women in Air Quality in South Asia, New Delhi 110088, India; ● Qatar Environment and Energy Research Institute, Environment & Sustainability Center, Doha, Qatar; ○ Kigali Collaborative Research Center, Carnegie Mellon Africa Campus, Kigali Innovation Center, Kigali, Rwanda; ■ People’s Collective for Environmental Justice, 224400 Barton Road, #21-296, Grand Terrace, California 92313, United States; □ Lamont Doherty Earth Observatory, Columbia University, 61 Route 9W, Palisades, New York 10964, United States; ▲ NASA Goddard Institute for Space Studies, 2880 Broadway, New York, New York 10025, United States; △ Department of Civil, Environmental, and Construction Engineering, University of Central Florida, 12760 Pegasus Drive, Orlando, Florida 32816, United States

**Keywords:** low-cost monitoring, community engagement, cultural competency, open access, Global South

Over the past several years, the advancement of low-cost air quality monitoring technologies has expanded the scope and scale of air quality research and has provided new opportunities for municipalities and community-led organizations to actively monitor air quality in a manner independent of regulatory agencies. Largely scaled up by private manufacturers, low-cost sensor (LCS) accessibility for monitoring criteria pollutants enables access to near-real-time data for individuals and organizations seeking to create or expand public awareness, advocate for improved air quality, support emissions reductions plans, and identify air pollution sources and hot spots. Many researchers and other interested parties have adopted LCS-based monitoring for their projects, more often with the goal of establishing spatially dense networks of LCS. Manufacturers, such as Atmos (India) and Clarity Movement (United States), commonly provide an accessible user interface for probing near-real-time measurements both spatially and temporally, allowing for unprecedented response times for generating answers to scientific inquiries.

The challenge that has been observed in this space regarding independently managed LCS networks is the choice of whether to make data publicly available and what temporal granularity of data should be made available for public consumption when the choice to share is available. One reason as to why academic researchers may be reluctant to make data public is the risk of “scooping” by well-established, better-resourced researchers who may take advantage of the opportunity to publish scientific findings and insights from a brand new, open-access data set, even when those researchers may not be involved in network creation and management. Academia rewards those who are “first to publish” and, as such, may tacitly incentivize use of openly available data, regardless of the means by which methods are developed or data are procured. Being first to publish new data leads to more citations, higher impact indices, and greater notoriety, in general, but therein lies an ethical dilemma that sits at odds with this model for academic research success. Another reason for reluctance in making LCS data public is the risk of limited or less accurate interpretation, for instance, in locations with dynamic meteorology and heterogeneous sources. In this case, LCS data corrections involve highly technical quantitative analysis, which may not be feasible for local network managers and community scientists. In addition, the lack of adequate local knowledge and information on the sensor network and its best correction method/model can lead to significant bias in the results. Even quality-controlled, corrected data would require local technical and scientific contributions to make the data worthy of peer review publication.

Additionally, LCS networks are usually established to monitor in areas that are chronically under-resourced and suffer from disproportionate environmental harms and injustices. Enabling the residents and other interested groups in these areas to manage, curate, and publish the data from their networks supports local campaigns to reverse these injustices. The aforementioned traditional academic practices become problematic when data are interpreted in ways that work against local goals and conclusions are drawn without due consideration of local knowledge or values. It has also been frequently noted among researchers in the Global South that data collected on environmental challenges from their jurisdiction are often published without any representation whatsoever of local experts as authors, a phenomenon termed helicopter or parachute science. This research is also often conducted without respect for local customs and values by Global North researchers. The natural response to prevent these practices, in the case of data from local LCS networks, is to restrict data access until local researchers and stakeholders are able to publish their findings with adequate representation. The lag in public availability of these data, which is sometimes on the order of years, limits knowledge generation with a threat to environmental and public health.

We propose the following solutions to address these challenges. First, environmental research journals could provide much-needed leadership toward the proposed goals by ensuring diversity in their editorial boards. Often these journals have little or no representation from scientists from the Global South, especially the African region, and the lack of representation should be addressed. In addition, environmental research journals already require a statement about research ethics. This could be expanded to include a statement of cultural competency when seeking to publish journal articles that feature locally generated data sets, especially those in underserved, underrepresented, or Global South communities. A cultural competency statement indicates the length of time that the research team has engaged with said community, the relationships established to successfully gather those data, and a statement of local challenges that the paper seeks to address. Thus, societies like the American Chemical Society and their editors can lead and actively support the push toward prioritizing (1) local and community data sovereignty and (2) the health and well-being of the communities that use research to advocate and improve their quality of life. The cultural competency statement functions similarly to an environmental significance statement, often required with manuscript submissions. This statement has also been recently successfully implemented as a requirement in the 2021–2022 research proposal solicitation from the California Air Resources Board (United States). Recently, *Nature* journals have also begun to encourage authors to follow the recommendations of the Global Code of Conduct for Research in Resource-Poor Settings in the design, execution, and reporting of research.

Second, global funding agencies could increase funding targeted at Global South researchers to enable them to be at the forefront of investigations on their local environmental challenge. To the extent that funding agencies and environmental societies in the Global North believe in producing the best possible science, their incentive to fund Global South researchers and build capacity for Global South researchers should be simply producing the best possible science. Without a Global South perspective on Global South environmental issues, the resulting research will be lacking, and any proposed solutions to an environmental problem will likely be ineffective. For example, local knowledge about sources of pollution can be critical for source apportionment and is not something that Global North researchers would readily know.

Third, professional environmental societies could institute programs that build the research capacity of Global South researchers to be able to conduct regional and globally relevant research and seek their publication in high impact factor journals for widespread visibility. Environmental societies already have the capacity and the resources to support the strengthening of technical know-how in the Global South. For example, the International Society of Exposure Science (ISES) and the American Association for Aerosol Research (AAAR) both organize preconference tutorials. Creating accessible opportunities to allow researchers from low- and middle-income countries to participate in such trainings can be an effective intervention. Also, capacity building targeted toward skill sets, such as effectively writing scientific, peer-reviewed journal articles and writing highly competitive major proposals, will help level the playing field. For example, other organizations, such as the International Society for Environmental Epidemiology (ISEE), now offer manuscript editing services to members with the goal of increasing acceptance rates of high-quality research manuscripts from researchers affiliated with institutions residing in low- and middle-income countries and those from underrepresented racial minority groups.

Lastly, researchers must continue to establish their overall aims of engagement with local communities. Outlining the objectives, goals, and aims is helpful for shaping the research project, as well as the plan for the dissemination of data. For example, researchers could ask, “Is the aim to establish a long-term monitoring program in an underserved community, or is the aim to highlight historical injustices of a proposed or long-established policy?” How researchers intend to engage the community in the long term will empower the community in their advocacy of environmental equity and develop long-term partnerships and thereby affect change that goes beyond the accomplishment of an academic publication.

In summary, we must continue to push toward local and community data sovereignty and the health and well-being of the communities that use research data to advocate and improve their quality of life. We continue to support ongoing efforts to promote knowledge transfer among Global South, underserved, or underresourced communities. Supporting local knowledge and open data access in such communities is already demonstrated in monitoring efforts around the world, including AfriqAir,^1^ GHAir,^2^ SAMOSA,^3,4^ and Belmont (Ohio, United States),^5^ as well as in open-access publishing, such as the new PLOS policy on inclusion in global research. Encouraging the publishing of locally generated data sets in the appropriate repositories also builds research capacity, increases the visibility of contributions from the Global South contributions, and broadens the pathway to gain citations for those contributions.^1^ We also recognize the importance of source apportionment applications and discussions about health and policy implications, which requires genuine partnerships with experts in these areas. Partnerships can provide the necessary reference instruments and filter-based monitoring that is necessary for source apportionment. We hope that our recommendations will further support local research efforts, leading to the development and implementation of specific policies to prioritize the health and well-being of local communities around the world.
